# Schizophrenia EEG Signal Classification Based on Swarm Intelligence Computing

**DOI:** 10.1155/2020/8853835

**Published:** 2020-11-30

**Authors:** Sunil Kumar Prabhakar, Harikumar Rajaguru, Sun-Hee Kim

**Affiliations:** ^1^Department of Brain and Cognitive Engineering, Korea University, Anam-dong, Seongbuk-gu, Seoul 02841, Republic of Korea; ^2^Department of Electronics and Communication Engineering, Bannari Amman Institute of Technology, Sathyamangalam 638402, India

## Abstract

One of the serious mental disorders where people interpret reality in an abnormal state is schizophrenia. A combination of extremely disordered thinking, delusion, and hallucination is caused due to schizophrenia, and the daily functions of a person are severely disturbed because of this disorder. A wide range of problems are caused due to schizophrenia such as disturbed thinking and behaviour. In the field of human neuroscience, the analysis of brain activity is quite an important research area. For general cognitive activity analysis, electroencephalography (EEG) signals are widely used as a low-resolution diagnosis tool. The EEG signals are a great boon to understand the abnormality of the brain disorders, especially schizophrenia. In this work, schizophrenia EEG signal classification is performed wherein, initially, features such as Detrend Fluctuation Analysis (DFA), Hurst Exponent, Recurrence Quantification Analysis (RQA), Sample Entropy, Fractal Dimension (FD), Kolmogorov Complexity, Hjorth exponent, Lempel Ziv Complexity (LZC), and Largest Lyapunov Exponent (LLE) are extracted initially. The extracted features are, then, optimized for selecting the best features through four types of optimization algorithms here such as Artificial Flora (AF) optimization, Glowworm Search (GS) optimization, Black Hole (BH) optimization, and Monkey Search (MS) optimization, and finally, it is classified through certain classifiers. The best results show that, for normal cases, a classification accuracy of 87.54% is obtained when BH optimization is utilized with Support Vector Machine-Radial Basis Function (SVM-RBF) kernel, and for schizophrenia cases, a classification accuracy of 92.17% is obtained when BH optimization is utilized with SVM-RBF kernel.

## 1. Introduction

The reason for schizophrenia occurrence is generally not known, but researchers believe that it is a combination of the environment and brain genetics which contributes a lot to the development of this disorder [1]. The signs and symptoms usually involve disorganized speech, delusions, impaired functions of organs, and hallucinations [2]. Symptoms generally vary with type and severity depending on time, sometimes remission of symptoms can occur and sometimes the existing symptom can worsen to a great extent. The symptom of schizophrenia in teenagers is more or less the same as those in adults as they experience a drop in performance at school, lack of motivation, irritability, depressed mindset, withdrawal from friends and family, and also suffer from trouble sleeping [3]. People affected with schizophrenia generally lack awareness that their difficulty originates from a mental disorder which requires careful medical screening [4]. Suicidal thoughts and behaviour too are very common symptoms of schizophrenia. Certain naturally occurring brain channels such as neurotransmitters when altered or disturbed may contribute to schizophrenia [5]. Though the precise cause of schizophrenia is not known, the common risk factors are having a family history of schizophrenia, birth complications, exposure to toxic elements, malnutrition, and psychotropic drugs which alter the state of mind [6]. If schizophrenia is left untreated, then it can result in a plethora of problems such as anxiety disorder, depression, alcohol abuse, social isolation, aggressive behaviour leading to victimization, social isolation, and financial issues followed due to various health and medical problems [7]. There is definitely no sure method to prevent schizophrenia, but considering and taking the treatment plan effectively can help in preventing relapses [8]. Characterized by relapsing episodes of psychosis, it is a serious disorder/mental illness. To determine the neural dynamics of human cognition, EEG recording acts as a sensitive tool as it can provide a millisecond-level resolution [9]. EEG data are complex, and at the same time, they are dimensional too as they are dependent on an event of time series [10]. As EEG signals provide electrical activity of the brain, it is easy to analyze the schizophrenia patient data.

Some of the most common works related to schizophrenia EEG signal analysis and classification reported in the literature are as follows. An EEG-dependent nonlinearity analysis technique for schizophrenia diagnosis was conducted by Zhao et al. [11]. The abnormal EEG complexity in patients with schizophrenia and depression was performed by Li et al. [12]. Fractal dimension was used by Raghavendra et al. [13] to analyze the complexity of EEG in schizophrenia patients. The complexity measures and entropy for EEG signal classification of both schizophrenia and control participants were performed by Sabeti et al. [14]. A preliminary report on the reduced nonlinear complexity and chaos during sleep in the first episode schizophrenia was given by Keshavan et al. [15]. A machine learning-based diagnosis of schizophrenia using combined sensor-level and source level EEG features was proposed by Shim et al. [16]. A preliminary data analysis of comparing the EEG nonlinearity in deficit and nondeficit schizophrenia patients was conducted by Cerquera et al. [17]. The nonlinear analysis of EEG in schizophrenia patients with persistent auditory hallucination was performed by Lee et al. [18]. For a schizophrenia patient, the estimate of the first positive Lyapunov exponent of the EEG signal was performed by Kim et al. [19]. A magnetoencephalography (MEG) study on the LZC in schizophrenia patients was conducted by Fernandez et al. [20]. A multiscale entropy analysis for the abnormal EEG complexity signals was performed by Takahashi et al. [21]. As a diagnostic test for schizophrenia, the spectral EEG abnormality was analyzed by Boutros et al. [22]. An in-depth analysis of the utility of quantitative EEG in unmedicated schizophrenia was conducted by Kim et al. [23]. For schizophrenic and healthy adults, the machine learning identification of EEG features which helps in predicting working memory performance was done by Johannesen et al. [24]. A data-driven methodology for resting state EEG signal classification of schizophrenia with control participants using random matrix theory was developed by Liu et al. [25]. Deep-learning methods along with random forest and voting classifiers was performed by Chu et al. for the individual recognition in schizophrenia using resting-state EEG streams [26]. Convolution Neural Networks (CNNs) along with the Pearson Correlation Coefficient (PCC) to represent the EEG channel relationships were used to classify the schizophrenic and healthy patients using EEG signals by Naira and Alamo [27]. Random Forest Machine learning algorithm was used to identify and diagnose the schizophrenia EEG signals by Zhang [28]. A higher order pattern discovery was used to classify the schizophrenia EEG by Sheng et al. [29]. The complexity of the EEG signals in schizophrenia syndromes was analyzed by Kutepov et al. [30]. A fractal-based classification of EEG signals for both schizophrenic and healthy patients was performed by Namazi et al. [31]. A new approach for EEG signal classification using Linear Discriminant Analysis (LDA) and Adaboost was found for schizophrenic and control participants by Sabeti et al. [32]. In this work, with the advent of some features, optimization techniques and classifiers, the schizophrenia EEG signal classification is performed, and this attempt is the first of its kind in schizophrenia EEG signal classification. The organization of the work is as follows.  Section 2 explains the materials and methods, Section 3 explains about the feature extraction techniques, Section 4 explains about the swarm intelligence computing techniques, Section 5 explains about the classification techniques, Section 6 explains the results and discussion followed by the conclusion in Section 7.

## 2. Materials and Methods

**Figure 1:**
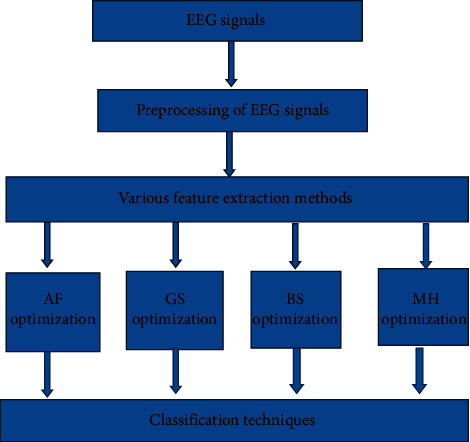
Pictorial representation of the work.

The EEG dataset for 14 healthy subjects and 14 Schizophrenic subjects was collected from the Institute of Psychiatry and Neurology, Warsaw, Poland, and the details are explained clearly in [33]. For the subjects, 7 males with an average age of 27.9 + 3.3 years and 7 females with an average age of 28.3 + 4.1 years were selected. The standard 10–20 International system was used for the acquisition of the EEG data. The patients' data were obtained in a relaxed state and with their eyes closed. The segmentation of the acquired EEG signals was performed where it is considered to be stationary. A very simplified pictorial representation of the work is given in Figure 1.

Over the duration of 15 minutes, 19 channel EEG signals are obtained. Every channel of EEG signals comprises of 2,25,000 samples which are, then, divided into groups of 5000 sample segments. Therefore, the data matrix of [5000 × 45] is framed per channel. For the preprocessing of EEG signals, Independent Component Analysis (ICA) was utilized in this work.

## 3. Feature Extraction Techniques

To describe a very large amount of data, feature extraction is necessary as it involves in mitigating the number of resources to a certain limit. Once the preprocessing of the EEG signals is conducted, the following features are extracted from the EEG signals as follows:

- DFA: to trace the self-similarity characteristics of the EEG signal, DFA [34] is used.
- Hurst exponent: the self-similarity and the predictability estimation of the EEG signal are expressed by the Hurst exponent [35]. If the magnitude value of the Hurst exponent is greater, then it denotes that the EEG signal is pretty smooth and less complicated.
- RQA: to measure the complexity of the EEG signals, the total number of times of recurrences is evaluated by RQA [36].
- Entropy: to evaluate the irregularity and uncertainty present in the EEG signal, entropy features are used. When the complexity and the variability of the EEG signal increases, then the entropy of the EEG signals is higher. Sample entropy [37], Shannon entropy [38], approximate entropy [39], Renyi entropy [40], permutation entropy [41], Kolmogorov–Sinai entropy [42], and fuzzy entropy [43] are the types of entropy usually used for the analysis, and in this work, only sample entropy has been extracted.
- FD: to compare and analyze the complexity of details in the EEG, FD is used, thereby enabling the detection of EEG signal patterns [44]. The Fractal Dimension of a signal is expressed through the Katz method as follows:

(1) $$\begin{array}{c} D=\frac{\log_{10}\left(H\right)}{\log_{10}\left(d\right)}, \end{array}$$

  where between the successive points, *H* represents the sum of distances and *d* represents the diameter estimated.
(vi) Kolmogorov complexity: the characteristics of the EEG signal are easily explained by this parameter [45]. If the signals are more random, then the description length is also longer.
(vii) Hjorth: to quantify the morphology of the EEG signal, the important Hjorth parameters utilized here are complexity, mobility, and activity [46].
(viii) LZC: to assess the repetitiveness of the EEG signals, LZC is used [47]. If the LZC values are higher, then it shows that the signal is more repetitive.
(ix) LLE: to assess the degree of chaos present in the EEG signals, an estimate of it is located by the LLE [48]. If the complexity of the signals is high, then the value of LLE is also high.

The feature extraction is initiated using DFA at first among the nine features. The attained feature matrix per method per channel is in the form of [5000 × 10]. Then, four types of optimization producer such as AF optimization, GS Optimization, BH optimization, and MS optimization are utilized to further extract a better represented feature column matrix as [5000 × 1]. This procedure is repeated for all the channels among the subjects. This feature extraction method is repeated for all the other eight features such as the Hurst exponent, RQA, entropy, fractal dimension, Kolmogorov complexity, Hjorth, and LLE, and thus, the feature extraction is performed for each data segment as such.

## 4. Swarm Intelligence Computing Techniques

To determine and understand a certain subset of initial features, feature selection is required. The features which are selected will usually have the useful and most relevant information from the original data so that, using the reduced form of representation, the desired task can be performed easily. The following four optimization techniques are utilized in this work.

### 4.1. Artificial Flora Algorithm

Developed by Cheng et al. [49], four basic elements are comprised in this algorithm such as original plant, offspring plant, location of the plant, and the distance of propagation initially. The plants that are used to spread seeds are called original plants. The seeds of original plants are called offspring plants, and at that moment, they cannot spread seeds. Plant location is the specific location of the plant, and the distance of propagation refers to how long a seed can spread. The three major patterns are present here such as evolution behaviour, spread behaviour, and select behaviour. The probability that the evolvement of the plant adapts to the environment behaviour is called evolution behaviour. The movement of seeds is referred by the spreading behaviour, and select behaviour refers to the survival or death of the flora due to the environment. The main rules here are as follows:

**Rule 1 .**

Due to the environmental or any external factors, a random distribution of a species in a region is performed; in that region, no such species were found earlier, and so, it becomes the primitive original plant.

**Rule 2 .**

As the environment changes, the plants will adopt to live in the main environment. Therefore, a complete inheritance to the parent plant does not depend on the proper distance of offspring plants.

**Rule 3 .**

When the seeds are spread around the original plant autonomously, the range is a circle where radius is the maximum propagation distance. Anywhere in the circle, the offspring plants can be distributed.

**Rule 4 .**

Plants have varied survival probability because of environmental factors such as climate and temperature. The survival probability is related to the fitness of the plants. Here, the adaptability of the plants to the environment can be referred by fitness. Or in other words, the survival probability of a plant in a specific position is termed as fitness. If the fitness is higher, then the probability of survival is greater.

**Rule 5 .**

The survival probability will be lower if the distance is further from the original plants because the basic difference between the current environment and the previous environment will be much greater.

**Rule 6 .**

If the seeds are spread in an external margin, then the spread distance cannot cross the maximum area of limit because of other constraints.

#### 4.1.1. Evolution Behaviour

The seeds are spread by the original plant around in a circle which is nothing but the propagation distance and is evolved for the respective propagation distances of the parent plant and the grandparent plant and is represented as

(2) $$\begin{array}{c} d_{h}=d_{1h}\times \text{rand}\left(0,1\right)\times c_{1}+d_{2h}\times \text{rand}\left(0,1\right)\times c_{2}, \end{array}$$

where *d*_1*h*_ is the propagation distance of the grandparent plant, *d*_2*h*_ is the propagation distance of the parent plant, *c*_1_ and *c*_2_ are the learning coefficients, and rand(0,1) denotes the independent uniformly distributed number in (0, 1).

The normal grandparent distance of propagation is expressed as

(3) $$\begin{array}{c} d_{1h}^{\prime }=d_{2h}. \end{array}$$

The standard deviation between the respective position of the original and offspring plant is the new parent propagation distance and is given as

(4) $$\begin{array}{c} d_{2h}^{\prime }=\sqrt{\frac{\sum_{i=1}^{N}{\left(L_{i,h}-L_{i,h}^{\prime }\right)}^{2}}{N}}. \end{array}$$

#### 4.1.2. Spreading Behaviour

The original flora with *N* solutions is randomly generated by the AF algorithm if there are *N* plants in the flora. The matrix *L*_*i*,*h*_ is used to express the original plant position where *i* is the dimension and *h* is the total number of plants in the flora.

(5) $$\begin{array}{c} L_{i,h}=\text{rand}\left(0,1\right)\times d\times 2-d, \end{array}$$

where the maximum limit area is represented by *d*, and rand(0,1) is an array of random numbers that have a uniform distribution between (0, 1). With the help of the following propagation function, the position of the offspring plant is generated as follows:

(6) $$\begin{array}{c} L_{i,h\times m}^{\prime }=D_{i,h\times m}+L_{i,h}, \end{array}$$

where the number of seeds that one plant can propagate is expressed as *m*, *L*_*i*,*h*×*m*_′ denotes the offspring position, *L*_*i*,*h*_ is the original plant position, and *D*_*i*,*h*×*m*_ is random number with the Gaussian distribution with zero mean and variance *d*_*h*_. If the survival of the offspring plant is not guaranteed, then a new plant is generated as shown in the abovementioned equation.

#### 4.1.3. Select Behaviour

The survival probability is used to assess whether the offsprings are alive or not and is represented as

(7) $$\begin{array}{c} l=\left|\sqrt{\frac{F\left(L_{i,h\times m}^{\prime }\right)}{F_{\max}}}\right|\times P_{y}^{\left(h\times m-1\right)}, \end{array}$$

where *P*_*y*_^(*h* × *m* − 1)^ is *P*_*y*_ to the power of (*h* × *m* − 1) and *P*_*y*_ is the selective problem. This value has to be between 0 and 1, and in our experiment, it is 0.5. When the offspring plant is farther from the original plant, then fitness is lower. *P*_*y*_ can assess the exploration ability of this algorithm. To get a local optima solution, *P*_*y*_ should be larger. The maximum fitness in the flora is determined by the *F*_max_, and the fitness of the *h*^th^ solution is determined by *F*(*L*_*i*,*h*×*m*_′). To decide whether the offspring plant is alive or not, the roulette wheel selection method is used in our work. The procedure is explained in Pseudocode 1.

*Pseudocode 1.*

  Input: times: maximum run time
   *B*: maximum branching number
   *N*: number of original plants
   *l*: survival probability of offspring plants
   *t*=0: population Initialization.
  Define related parameters
  Evaluate the fitness value of *N* individuals and compute the best solution
  While (*t*<times)
   For *i*=1 : *N∗B*
    Propagation distance is evolved by new plants
    Original plants spread their offspring
    If rand(0,1) > *l*
     Offspring is alive
    Else
    Offspring is dead
    End if
   End for
   Evaluate novel solutions and randomly select *N*  plants as new original plants
   If new solution is better, then old plant is replaced by new plant
   Find the current best solution
   *t*=*t*+1
  End while
  Output: optimized solution

### 4.2. Glowworm Swarm Optimization Algorithm

In this algorithm, initially, the position location and information on data exchange can be carried out by most glowworms by means of sending out rhythmed short beams [50]. Around a particular searching scope, the main intention of GS optimization is to find out the flaring neighbor. The glowworms always move from the first position to a best position and finally into a more extreme value point. The attraction of the glowworm individuals is highly related to its brightness. The attractiveness of a particular individual glow worm is inversely proportional to the distance between the two individuals, so it implies that it has a direct proportion to brightness. Each position of the individual glow worm accounts for the objective function value. The individual search scope is defined by the dynamic decision domain. The individual movement is later updated step by step in the procedure given below.

#### 4.2.1. Procedure

- Parameter initialization: ′*i*′ individuals are initially placed in a random fashion around a feasible region. *f*_0_ denotes the fluorescein value, *q*_0_ indicates the dynamic decision domain, st indicates step, *i*_*n*_ expresses domain threshold, the update coefficient of domain is expressed as *β*, *q*_st_ denotes the maximum search radius, and the iteration number is expressed as *n*.
- The objective function value *H*(*y*_*j*_(*n*)) is transformed to *f*_*j*_(*n*) as

(8) $$\begin{array}{c} f_{j}\left(n\right)=\left(1-\rho \right)f_{j}\left(n-1\right)+\gamma H\left(y_{j}\left(n\right)\right), \end{array}$$

  where *y*_*j*_(*n*) expresses for the individual position *j* at ′*n*′ instant of time.
(iii) In each *q*_*d*_^*j*^(*n*), the higher fluorescein value is selected, thereby forming a set of neighborhood *I*_*j*_(*n*). Therefore,

(9) $$\begin{array}{c} I_{j}\left(n\right)=\left\{h:\left\|y_{h}\left(n\right)-y_{j}\left(n\right)\right\|<q_{d}^{j}\left(n\right);f_{j}\left(n\right)\leq f_{h}\left(n\right)\right\}. \end{array}$$

(iv) The probability of a particular individual *j* may progress forward as *h* and is expressed as

(10) $$\begin{array}{c} p_{jh}\left(n\right)=\frac{f_{h}\left(n\right)-f_{j}\left(n\right)}{\sum_{m\in I_{j}\left(n\right)}f_{m}\left(n\right)-f_{j}\left(n\right)}, \end{array}$$

  where *h* is chosen by *p*_*jh*_(*n*).
(v) The updation of the position of individual *j* is expressed as

(11) $$\begin{array}{c} y_{j}\left(n+1\right)=y_{j}\left(n\right)+\text{st}\left(\frac{y_{h}\left(n\right)-y_{j}\left(n\right)}{\left\|y_{h}\left(n\right)-y_{j}\left(n\right)\right\|}\right). \end{array}$$

(vi) The updation of the dynamic decision domain is expressed as

(12) $$\begin{array}{c} q_{d}^{j}\left(n+1\right)=\min\left\{q_{\text{st}},\max\left\{0,q_{d}^{j}\left(n\right),\beta \left(i_{n}-\left|I_{j}\left(n\right)\right|\right)\right\}\right\}. \end{array}$$

### 4.3. Black Hole Algorithm

The algorithm is inspired from the black hole phenomenon [51]. In this method, at each iteration, the best candidate is chosen as the black hole and the other normal candidates are chosen as normal stars. The black hole creation is one of the original candidates of the entire population, and it is not random. Depending on one's current location and a randomly generated number, the movement of the candidate towards the black hole is ascertained. The step-by-step details of the algorithm is as follows:

- It is a very famous population-based algorithm. Therefore, in the search space of some function, some randomly generated population of candidate solutions (stars) is placed.
- The fitness value evaluation of the population is conducted after the initialization process.
- The best candidate in the population is the one which has the highest fitness value, and it is assumed to be the initial black hole.
- While this process is going on, the rest of the candidates do the normal stars.
- The specialty of the black hole is that it has the capacity to absorb the surrounding stars around it.
- Once the stars and the black hole are initialized, the absorption of the stars by the black hole takes place, and therefore, the movement of all other stars is now towards or around the black hole.
- The formulation of the capability of the absorption of stars by the black hole is ascertained as follows:

(13) $$\begin{array}{c} Y_{j}\left(t+1\right)=Y_{j}\left(t\right)+\text{rand}.\left(Y_{cl}-Y_{j}\left(t\right)\right). \end{array}$$

Here, *Y*_*j*_(*t*) and *Y*_*j*_(*t*+1) are the locations of the star *j* in iteration *t* and *t*+1. *Y*_*cl*_ is the location of the black hole in the entire search space. Random number in the interval of [0,1] is determined by rand.
(viii) The black hole absorbs the candidate solution (each star representing it) that crosses the horizon of the black hole. Using the following equation, the radius of the horizon in the BH algorithm is computed as follows:
  (14) $$\begin{array}{c} R=\left|\frac{G_{cl}}{{\sum_{j=1}^{S}G}_{j}}\right|, \end{array}$$

where *G*_*cl*_ represents the black hole fitness value, *G*_*j*_ is the fitness value of the star *j*, *S* denotes the number of stars which indicates the size of population/candidate solutions, and *R* denotes the black hole radius.

The candidate is usually collapsed when the distance between the best candidate (black hole) and the candidate solution is less than *R*. In such a case, the value of a new candidate would be carried out, and it is randomly distributed in the search space giving the optimized and the best values.

### 4.4. Monkey Search Algorithm

Based on the mountain climbing procedure of monkeys, one of the recently developed metaheuristic algorithms is MS algorithm [52]. Initially, for the monkeys, the population size is defined. Each monkey's specific position is denoted by a vector *a*_*i*_, and it is performed randomly. With the help of a step-by-step climbing process, the monkey positions are changed. One of the famous recursive optimization algorithm called Simultaneous Perturbation Stochastic Approximation (SPSA) was used to design the climb process in MS algorithm. The objective function value is improved by the climbing process. Once a monkey arrives on top of a mountain after the climbing process, then it will search for higher points than its current position. A monkey with its sharp and keen eyesight would easily shift its base to a higher point by jumping. The maximum distance watched by a monkey is determined by the eyesight of a monkey. The updation of the position is carried out. Then, the monkeys start to search the novel search domains by utilizing the current position as a pivot. This process is termed as a somersault procedure, and it helps the monkeys to get a new position. The evaluation of the objective function values is conducted, and if the number of iterations is satisfactory, then the process will be stopped. The main process of this algorithm is as follows:

(1) Representation of a solution: the population size of monkey *M* is defined initially. For any monkey, *j* ∈ {1,2,…, *M*} defines its position *a*_*j*_=(*a*_*j*1_, *a*_*j*2_,…, *a*_*jn*_) which gives the optimization problem solutions with the *n* dimensions.
(2) Initialization: in a random manner, the initial solution of the monkeys is generated.
(3) Climbing process:
  - A vector is generated randomly Δ*a*_*j*_=(Δ*a*_*j*1_, Δ*a*_*j*2_,…, Δ*a*_*jn*_), where Δ*a*_*jk*_ is set as *s*, where *s* denotes the step length of climb process.

(15) $$\begin{array}{c} g_{jk}^{\prime }\left(a_{j}\right)=\frac{g\left(a_{j}+\Delta a_{j}\right)-g\left(a_{j}-\Delta a_{j}\right)}{2\Delta a_{jk}}, \end{array}$$

 
*k*=1,2,…, *n*, and at point *a*_*j*_, the vector is termed as the pseudogradient of the objective function
(ii) Assign *z*_*k*_=*a*_*jk*_+*α* sign(*g*_*jk*_′(*a*_*j*_)), *k*=1,2,…, *n* and *z*=(*z*_1_, *z*_2_,…, *z*_*n*_)
(iii) Assume *a*_*j*_ ← *z* has given *z* is feasible or else keep as such *a*_*j*_
(iv) Unless the maximum number of allowed iterations *N*_*q*_ has reached or there is a little change in the objective function of the iteration, the steps (i) to (iii) of the climbing process are repeated
(4) Watch jump process:
(i) The real number *z*_*j*_ is generated randomly from (*a*_*jk*_ − *e*, *a*_*jk*_+*e*), *k*=1,2,…, *n*, where *e* represents the eyesight of the monkey which denotes the maximum distance that can be witnessed by a monkey.
(ii) Assume *a*_*j*_⟵*z* given that *g*(*z*) > *g*(*a*_*j*_) and *z* is feasible. Unless a certain number of watch times has been obtained or until an appropriate point *z* is found out, the steps are repeated.
(iii) The climb process is repeated by employing *z* as an initial position.
(5) Somersault process:
(i) From the somersault interval [*q*, *d*], a real number *θ* is generated randomly.
(ii) Set *z*_*k*_=*a*_*jk*_+*θ*(*v*_*k*_ − *a*_*jk*_), where *v*_*k*_=(1/*M*)∑_*j*=1_^*M*^*a*_*jk*_,  *k*=1,2,…, *n*.
 
*v*=(*v*_1_, *v*_2_,…, *v*_*n*_) is termed somersault pivot and (*v*_*k*_ − *a*_*jk*_) is the somersault direction of monkey *j*.
(iii) Set *a*_*j*_ ← *z* if *z*=(*z*_1_, *z*_2_,…, *z*_*n*_) is quite feasible. Unless a feasible solution *z* is found out, steps (i) and (iii) of this somersault process are repeated.
(6) Termination.

Unless the stopping criterion is met, the abovementioned steps are repeated. As the stopping criteria, the number of iterations are used.

Thus, using these metaheuristic algorithms, the optimal and best solutions are explored and exploited from the initial random solutions.

## 5. Classification Techniques

The optimized values or the best selected feature values through swarm intelligence computing techniques are finally fed to the classifiers for classification.

### 5.1. ANN

Here, a three-layer perceptron comprising of an input layer, hidden layer, and an output layer was utilized [53]. The total number of hidden neurons is expressed by the following equation:

(16) $$\begin{array}{c} H=\left[\left(A+B\right)d\right], \end{array}$$

where the number of input neurons is expressed as *A*, the number of output neurons is expressed as *B*, and *d* is a constant and its range is from *d* ∈ [0,1]. For the ANN classifier, the total number of hidden neurons used here is 50.

### 5.2. QDA

The ratio of the between class variance is maximized, and the ratio of the within class variance is minimized by QDA [54]. Between classes, it also allows quadratic decision boundaries so that the classifier can perform the classification more accurately providing good classification accuracy. In this QDA, no shrinkage or regularization is used.

### 5.3. SVM

Due to its good scalability and its high classification performance, SVM is used [55]. Creating a hyperplane to maximize the margins between the classes that can be obtained by mitigating the cost function, so that the maximum classification accuracy is attained is the main idea of SVM. The hyperplanes which are represented by the vectors are known as support vectors. By minimizing the cost function, the optimal solution that maximizes the distance between the hyperplane and the nearest training point is obtained by the SVM as follows:

(17) $$\begin{array}{c} \text{Minimize,}\frac{1}{2}{\left\|w\right\|}^{2}+C\sum_{j=1}^{n}\xi_{j},\\ \text{subject to,}z_{j}{\left(w^{T}x_{j}+f\right)}^{3}\geq 1-\xi_{j},\xi_{j}\geq 0, \end{array}$$

where *w*^*T*^, *x*_*j*_ ∈ *R*^2^ and *f* ∈ *R*′, ‖*w*‖^2^=*w*^*T*^ *w*, 

The tradeoff between the margin and the error is denoted as *C*. The measure of the training data is expressed as *ξ*_*j*_ The class label for the *j*^th^ sample is denoted as *z*_*j*_. SVM can be utilized as a both linear and nonlinear classifier. Various types of kernel functions are utilized to make SVM as a nonlinear classifier. The types of kernels generally used are Polynomial, Radial Basis Function (RBF), and Sigmoid kernels. Here, in our work, only SVM-RBF kernel is used, and this nonlinear SVM is used to get higher classification accuracy.

### 5.4. Logistic Regression

Between an independent variable and a response variable, the relationship is assessed by Logistic Regression (LR) [56]. An observation is classified into one of the two classes using Logistic Regression in a very simple manner. When the variables are nominal or binary, it can be used. Similar to the Bayesian group, the data are comprehensively analyzed after the discretization process for the continuous variables is performed.

### 5.5. FLDA

The main intention of the Fischers Linear Discriminant Analysis (FLDA) is to trace a direction in the feature space along which the specific distance of the means relative to the within-class scatter explained by the within-class scatter matrix *S*_*W*_ reaches a maximum [57]. When the within-class scatter matrix reaches a maximum, the class separability is maximized. By maximizing the following criterion with the between-class scatter matrix, this goal can be achieved.

(18) $$\begin{array}{c} J\left(W\right)=\frac{W^{T}S_{B}W}{W^{T}S_{W}W}. \end{array}$$

To maximize the criterion, the direction *w* is expressed as *W*=*S*_*W*_^−1^(*m*_1_ − *m*_2_), where *m*_1_ and *m*_2_ are the means for the two classes. For the two classes, the FLDA acts as a suboptimal classifier when their respective distributions are Gaussian in nature.

### 5.6. KNN

One of the famous nonparametric algorithms utilized for both classification and regression is KNN [58]. On the underlying data distribution, KNN does not make any assumption. There is also no explicit training phase available here. During the testing phase, the utilization of the training data is carried out where the measurement between the training instance and test instance is performed. The prediction of the class of the test instance is performed by utilizing the majority voting of the any of the K-nearest training instances. The value of K is assumed to be 4 in our work.

## 6. Results and Discussion

It is classified with 70% of training and 30% of testing methodology and the performance of it is computed. The experiment was repeated five times to check whether we get the similar results every time when the analysis is done. The mathematical formulae for computing the Performance Index (PI), Sensitivity, Specificity, Accuracy, Good Detection Rate (GDR), and Mean Square Error (MSE) rate is mentioned in literature, and using the same, the values are computed and exhibited. PC indicates Perfect Classification, FA indicates False Alarm, and MC indicates Missed Classification, respectively.

The sensitivity is expressed as

(19) $$\begin{array}{c} \text{Sensitivity}=\frac{\text{PC}}{\text{PC}+\text{FA}}\times 100. \end{array}$$

Specificity is expressed as

(20) $$\begin{array}{c} \text{Specificity}=\frac{\text{PC}}{\text{PC}+\text{MC}}\times 100. \end{array}$$

Accuracy is expressed as

(21) $$\begin{array}{c} \text{Accuracy}=\frac{\text{Sensitivity}+\text{Specificity}}{2}. \end{array}$$

Performance Index is expressed as

(22) $$\begin{array}{c} \text{PI}=\left(\frac{\text{PC}-\text{MC}-\text{FA}}{\text{PC}}\right)\times 100. \end{array}$$

Good Detection Rate (GDR) is expressed as

(23) $$\begin{array}{c} \text{GDR}=\left(\frac{\left[\text{PC}-\text{MC}\right]}{\left[\text{PC}+\text{FA}\right]}\right)\times 100. \end{array}$$

The Mean Square Error (MSE) is expressed as follows:

(24) $$\begin{array}{c} \text{MSE}=\frac{1}{N}\sum_{i=1}^{N}{\left(O_{i}-T_{j}\right)}^{2}, \end{array}$$

where *O*_*i*_ indicates the observed value at a specific time, *T*_*j*_ denotes the target value at model *j*; *j* = 1 to 19, and *N* is the total number of observations per channel for a single optimization method, and in our case, it is 45000. The training of the classifiers was implemented with a zero-training error of MSE.

Table 1 shows the average statistical parameters such as mean, variance, skewness, and kurtosis of the nine extracted features through four-optimization process for the normal cases. The higher value of mean indicates the peaked position of the feature selected. Lower value of mean indicates that there exists the peak and valley points in the features. As in the case of AF optimization in Kolmogorov complexity features, peaked value of mean is attained. The variance parameter shows the energy component of the feature. Here also in Kolmogorov complexity in AF optimization method arrived higher value. Skewness depicts the skewed features of the data points, and all the features in Table 1 indicates the same. The flatness is indicated by the higher kurtosis values. In the case of Hurst exponent, Fractal dimension and Hjorth features at AF optimization show higher value of kurtosis.

Table 2 demonstrates the average statistical parameters such as mean, variance, skewness, and kurtosis of the nine extracted features through four-optimization process for the schizophrenia cases. The higher value of mean indicates the peaked position of the feature selected. Lower value of mean indicates that there exist the peak and valley points in the features, as all the optimization methods made the mean parameter as a smaller one among the nine features. Skewness depicts the skewed features of the data points and all the features in Table 2 indicate the same. The flatness is indicated by the higher kurtosis values. In the case of Kolmogorov complexity at AF optimization and BH optimization, it shows higher value of Kurtosis.

The correlation among the normal and schizophrenia cases can be established by calculating the Canonical Correlation Analysis (CCA) as shown in Table 3. If the CCA value is greater than 0.5 that indicates the two classes are highly correlated and for lower value it is vice versa. As shown in Table 3 the CCA is calculated for the nine features among the normal and schizophrenia cases. As observed from Table 3, the lower value of CCA indicates the Nil correlation among the features across the classes.

Table 4 exhibits the average PCC with different features for normal and schizophrenia cases. The values in Table 2 indicates the nonlinear relation among the features in the same class of the data. Therefore, the uncorrelated and nonlinear features have to be optimized by the optimization process.

Table 5 shows the CCA of various optimization techniques with different features for normal and schizophrenia cases. It is observed from Table 5 that the low value of CCA is definitely indicating the Nil correlation among the features of the two classes.

Table 6 depicts the Average PCC at various optimization techniques with nine different features for normal and schizophrenia cases. As shown in Table 6, low value of CCA in the normal cases indicates the presence of nonlinearity in the features. The negative value of PCC in the schizophrenia cases mention about the inverse relation among the features as well as the optimization methods.

Table 7 shows the consolidated results of accuracy among the classifiers at various optimization techniques with different features of normal cases. As indicated in Table 7, ANN classifier is ebbed at low value of accuracy in the three types of optimization methods such as AF, GS, and BH. The poor performance of ANN is due to over learning and the exhibition of false alarm in the classifier outputs. FLDA classifier arrived at low accuracy in the case of MS optimization method. SVM classifier is outperforming all the classifiers in terms of higher accuracy value of 87.54% in BH optimization method.

Table 8 denotes the consolidated results of accuracy among the classifiers at various optimization techniques with different features of schizophrenia cases. As observed in Table 8, LR classifier is ebbed at low value of accuracy of 78.64% in the black hole optimization method. The poor performance of LR is due to the rigid parametric values and the exhibition of missed classification in the classifier outputs. SVM classifier is outperforming all the classifiers in terms of higher accuracy value of 92.17% in BH optimization method. Evenly nature of the performance shows higher accuracy values among the classifiers for MS method.

Table 9 represents the average perfect classification among the classifiers at various optimization techniques with different features of normal cases. As observed in Table 9, LR classifier is ebbed at low value of perfect classification of 54.3% in the AF optimization method. The poor performance of LR is due to the nonadaptive parametric values and the exhibition of false alarm in the classifier outputs. SVM classifier is outperforming all the classifiers in terms of higher perfect classification value of 75.093% in BH optimization method. Evenly nature of the performance shows higher perfect classification values among the classifiers for MS method.

Table 10 denotes the average perfect classification among the classifiers at various optimization techniques with different features of schizophrenia cases. As shown in Table 10, LR classifier is reached at low value of perfect classification of 57.29% in the BH optimization method. The poor performance of LR is due to the nonadaptive parametric values and the exhibition of missed classification in the classifier outputs. SVM classifier is outperforming all the classifiers in terms of higher perfect classification value of 84.34% in BH optimization method. Evenly nature of the performance shows higher perfect classification values among the classifiers for MS method.

Table 11 depicts the average Performance Index among the classifiers at various optimization techniques with different features of normal cases. As shown in Table 11, LR classifier is reached at low value of PI of 15.63% in AF optimization method. The poor performance of LR is due to the exhibition of missed classification in the classifier outputs. SVM classifier is outperforming all the classifiers in terms of higher PI value of 64.6% in BH optimization method. The performance of PI parameter for the ANN and QDA classifier among the four-optimization method is poor, and this is due to the more missed classification and false alarms of the classifier outputs.

Table 12 signifies the average Performance Index among the classifiers at various optimization techniques with different features of schizophrenia cases. As shown in Table 12, LR classifier is reached at low value of PI of 24.1% in the BH optimization method. The poor performance of LR is due to the exhibition of missed classification in the classifier outputs. SVM classifier is outperforming all the classifiers in terms of higher PI value of 81.59% in BH optimization method. Once again, MS optimization evenly handled the PI parameter among the classifiers.

Table 13 exhibits the average performance of parameters among the classifiers at various optimization techniques with different features of normal cases. As shown in Table 13, ANN classifier is reached at low value of PI of 22.01 and PC of 56.7%. FLDA denotes low GDR value of 37.67%. SVM classifier is outperforming all the classifiers in terms of higher PC, PI, and GDR values and lower error rate value of 27.4%. For a significant accuracy and error rate, KNN classifier closely follows the performance of the SVM classifier.

Table 14 depicts the average performance of parameters among the classifiers at various optimization techniques with different features of schizophrenia cases. As shown in Table 14, LR classifier is reached at low value of PI of 31.5% and PC of 60.67%. LR denotes low GDR value of 41.9%. SVM classifier is outperforming all the classifiers in terms of higher PC, PI, and GDR values and lower error rate value of 18.11%. For a significant accuracy and error rate, ANN classifier closely follows the performance of the SVM classifier.

## 7. Conclusions and Future Work

The disorders in the areas of the lobes of the brain can lead to schizophrenia. As the lobes of the brain are important for information processing and memory management activities, a huge damage occurs to it due to schizophrenia. The diagnosis, classification, and analysis of schizophrenia spectrum disorders are quite challenging. To incorporate the latest scientific techniques to clinical diagnosis, the scientific community is working very hard. For the brain state interpretation and diagnosis, EEG is emerged as a highly useful and beneficial tool. The proposed method in this work explores and utilizes a plethora of features with four different types of optimization techniques before proceeding to classification. The best results show that, for normal cases, a classification accuracy of 87.54% is obtained when BH optimization is utilized with SVM-RBF Kernel, and for schizophrenia cases, a classification accuracy of 92.17% is obtained when BH optimization is utilized with SVM-RBF kernel. We plan to incorporate other optimization mechanisms with deep learning techniques for schizophrenia EEG signal classification in future work.

## Figures and Tables

**Table 1:** Average statistical parameters at various optimization techniques with different features for normal cases.

Optimization methods	Parameters	DFA	Hurst	RQA	Sample entropy	Fractal dimension	Kolmogorov complexity	Hjorth	LZC	LLE
Artificial flora	Mean	5.26965	0.413804	3.689182	0.869676	0.41564	28.75062	0.4173837	0.416007	0.4172
	Variance	22.07468	0.000125	0.961903	1.100939	8.993E−06	311.8672	2.16155E−10	1.17142E−07	3.939E−08
	Skewness	1.724139	−7.92829	−0.98766	2.580366	−4.544	1.179709	−6.72855	1.5590	−3.7801
	Kurtosis	5.223521	77.60378	0.927814	9.156234	30.0779	2.794157	49.372894	2.6237	20.561

Glowworm swarm	Mean	2.320835	0.733608	0.396612	1.731146	0.74723	0.609228	0.196184	0.269188	0.1472
	Variance	1.03798	0.021621	0.001249	0.049742	0.019817	0.033745	2.14E−06	5.15E−06	0.00035
	Skewness	1.903065	0.269845	1.104594	−0.20551	0.240516	2.29958	0.698693	0.408151	1.02453
	Kurtosis	4.714625	0.122304	2.464601	0.171725	0.032322	8.864067	0.245379	8.982281	2.43118

Black hole	Mean	2.710718	0.351737	1.363744	2.227318	0.524266	3.234247	1.75846	1.61927	1.59492
	Variance	0.123383	0.044921	0.011921	0.524723	0.055867	0.173108	0.000821	0.005577	0.110766
	Skewness	0.55226	−0.2302	−0.41571	1.004853	−0.45211	0.861662	1.221792	−11.2091	0.540982
	Kurtosis	0.090556	0.133614	−1.56857	4.072836	0.456164	0.082189	−0.46675	226.0795	1.804519

Monkey search	Mean	0.444369	0.000265	0.044637	0.066802	0.000593	0.786268	0.556733	0.740261	0.708153
	Variance	0.008841	6.47E−08	7.64E−05	0.000456	3.51E−07	0.005208	0.001272	0.003981	67.04619
	Skewness	0.0065	2.394641	0.614119	0.430518	2.170684	−0.33499	0.572398	19.90603	16.253
	Kurtosis	−0.13621	8.868095	0.241653	0.532993	6.750117	0.005905	0.058357	687.3791	282.403

**Table 2:** Average parameters at various optimization techniques with different features for schizophrenia cases.

Optimization methods	Parameters	DFA	Hurst	RQA	Sample entropy	Fractal dimension	Kolmogorov complexity	Hjorth	LZC	LLE
Artificial flora	Mean	1.947	0.797	0.895	1.0434	0.9192	0.9182	0.922	0.922	0.72722
	Variance	6.1955	0.00198	8.215E−05	0.9472	7.331E−05	0.00011	4.82E−09	3.393E−08	0.00382
	Skewness	2.7591	−0.0952	0.1699	3.4882	−7.9156063	−31.6135	4.8058	1.162	0.66921
	Kurtosis	10.378	−0.3245	−0.00067	16.5549	139.9124	998.7822	25.231	2.795	0.34590

Glowworm swarm	Mean	3.60521	0.45140	0.528535	3.20544	1.109916	2.762614	0.775	0.874	0.43363
	Variance	1.40291	0.00050	0.000336	0.18103	0.019184	0.846133	0.00022	3.85E−06	0.000213
	Skewness	1.83474	0.18256	−0.0874	−0.02972	0.21627	1.943948	0.53419	−0.373	0.538756
	Kurtosis	4.37384	−0.12708	−0.09477	−0.32559	−0.17723	4.561639	0.0462	11.51	0.5087

Black hole	Mean	0.04486	1.64093	0.38062	0.08666	0.471683	0.193926	0.246	0.209	1.409073
	Variance	0.01454	0.827833	0.000476	0.020484	0.054866	0.00054	1.09E−05	6.7E−06	0.102673
	Skewness	−0.911996	2.338573	0.602759	−0.25059	2.264549	31.66492	−2.561	9.701	1.243274
	Kurtosis	1.689095	8.655974	0.923601	−1.38165	8.043591	1003.234	5.236	156.25	3.155236

Monkey search	Mean	0.360178	0.64032	0.142771	0.139355	0.000866	0.540915	0.933	0.000151	2.272099
	Variance	0.01044	0.449776	0.000665	0.001762	2.15E−07	0.006902	0.00703	1.9E−12	85.4282
	Skewness	0.232422	2.867251	0.441075	0.154336	0.980327	−0.20562	0.5348	0.799874	11.61515
	Kurtosis	−0.06264	13.54124	0.384905	−0.46198	1.1591	−0.30885	0.0581	10.16941	167.628

**Table 3:** Average CCA with different features for normal and schizophrenia cases.

Parameters	DFA	Hurst	RQA	Sample entropy	Fractal dimension	Kolmogorov complexity	Hjorth	LZC	LLE
CCA	0.14231	0.05738	0.0876	0.14356	0.06889	0.15469	0.14003	0.06534	0.12817

**Table 4:** Average PCC with different features for normal and schizophrenia cases.

Parameters PCC	DFA	Hurst	RQA	Sample entropy	Fractal dimension	Kolmogorov complexity	Hjorth	LZC	LLE
Normal	0.021077	0.014018	0.006344	0.047908	0.016744	0.023395	0.00045	0.00376	0.062845
Schizophrenia cases	0.053808	0.012278	0.068795	0.030514	0.032101	0.107226	0.003109	0.00216	0.043985

**Table 5:** CCA at various optimization techniques with different features for normal and schizophrenia cases.

Optimization methods	CCA
Artificial flora	0.047944
Glowworm swarm	0.088456
Black hole	0.060256
Monkey search	0.089556

**Table 6:** Average PCC at various optimization techniques with different features for normal and schizophrenia cases.

Optimization methods	PCC	
	Normal	Schizophrenia cases
Artificial flora	0.005745	−0.01573
Glowworm swarm	0.04604	−0.07745
Black hole	0.040539	−0.01178
Monkey search	0.08175	−0.1422

**Table 7:** Consolidated results of accuracy (%) among the classifiers at various optimization techniques with different features for normal cases.

Optimization methods	ANN	QDA	SVM	LR	FLDA	KNN
Artificial flora	77.42581	79.08989	85.40703	77.15035	80.07678	82.61267
Glowworm swarm	77.35069	79.03598	87.14997	83.3216	80.18607	81.87646
Black hole	77.25071	78.82409	87.54716	77.3041	80.27763	79.3533
Monkey search	81.47733	79.93699	85.08729	84.48008	79.67025	81.07509

**Table 8:** Consolidated results of accuracy (%) among the classifiers at various optimization techniques with different features for schizophrenia cases.

Optimization methods	ANN	QDA	SVM	LR	FLDA	KNN
Artificial flora	83.33014	79.66433	90.60692	79.11691	81.08746	79.24898
Glowworm swarm	83.95856	79.89032	89.62917	81.28533	80.95178	82.65979
Black hole	85.2518	79.5597	92.17549	78.64844	83.37886	79.64616
Monkey search	86.06456	82.1462	91.37198	82.34527	81.96181	83.82426

**Table 9:** Average perfect classification (%) among the classifiers at various optimization techniques with different features for normal cases.

Optimization methods	ANN	QDA	SVM	LR	FLDA	KNN
Artificial flora	54.85161	58.17979	70.81364	54.3007	60.15023	65.22285
Glowworm swarm	54.70138	58.07196	74.2991	66.63904	60.36965	63.75292
Black hole	54.50142	57.64817	75.09224	54.6082	60.55359	58.7066
Monkey search	62.95465	59.87399	70.17104	68.95516	59.34051	62.15018

**Table 10:** Average perfect classification (%) among the classifiers at various optimization techniques with different features for schizophrenia cases.

Optimization methods	ANN	QDA	SVM	LR	FLDA	KNN
Artificial flora	66.66027	59.32866	81.20947	58.23381	62.17283	58.49795
Glowworm swarm	67.91504	59.7798	79.25667	62.57066	61.90357	65.31875
Black hole	70.49922	59.11939	84.34931	57.29688	66.75354	59.29232
Monkey search	72.1262	64.2897	82.74271	64.68721	63.9228	67.64852

**Table 11:** Average performance index (%) among the classifiers at various optimization techniques with different features for normal cases.

Optimization methods	ANN	QDA	SVM	LR	FLDA	KNN
Artificial flora	17.53914	26.58122	57.531	15.63139	31.68778	43.07008
Glowworm swarm	17.00598	26.44067	63.01535	46.80996	31.16374	38.30346
Black hole	16.29544	25.40426	64.60384	16.62192	29.83996	27.25943
Monkey search	37.21904	30.80569	53.90694	51.9737	30.50127	34.76854

**Table 12:** Average performance index (%) among the classifiers at various optimization techniques with different features for schizophrenia cases.

Optimization methods	ANN	QDA	SVM	LR	FLDA	KNN
Artificial flora	47.33871	30.02955	76.75093	26.06912	34.70983	26.38648
Glowworm swarm	50.81356	29.57847	72.88667	34.9601	33.86657	41.54275
Black hole	54.83947	27.8239	81.59472	24.10137	45.32586	28.93259
Monkey search	59.19286	41.31742	78.69396	40.9822	42.56467	49.06799

**Table 13:** Average performance of parameters among the classifiers at various optimization techniques with different features for normal cases.

Performance parameters	ANN	QDA	SVM	LR	FLDA	KNN
Perfect classification	56.75227	58.44348	72.59401	61.12578	60.1035	62.45814
Performance index	22.0149	27.30796	59.76428	32.75924	30.79819	35.85038
Accuracy	78.37614	79.22174	86.29786	80.56403	80.05268	81.22938
GDR	54.51164	58.44344	72.43905	59.11251	37.6745	55.96857
Error rate	43.24759	41.55656	27.4062	38.87433	39.89642	37.54199

**Table 14:** Average performance of parameters among the classifiers at various optimization techniques with different features for schizophrenia cases.

Performance parameters	ANN	QDA	SVM	LR	FLDA	KNN
Perfect classification	69.30018	60.62939	81.88954	60.69714	63.68819	62.68939
Performance index	53.04615	32.18734	77.48157	31.5282	39.11673	36.48245
Accuracy	84.65127	80.31514	90.94589	80.34899	81.84498	81.3448
GDR	59.85653	60.18572	80.80855	41.90007	45.78074	52.10981
Error rate	30.7002	39.37056	18.11075	39.30309	36.31199	37.31102

## Data Availability

Data will be provided to genuine researchers upon request.
